# Intrathecal synthesis of IgE in children with eosinophilic meningoencephalitis caused by *Angiostrongylus cantonensis*

**DOI:** 10.1186/1743-8454-5-18

**Published:** 2008-11-25

**Authors:** Barbara Padilla-Docal, Alberto J Dorta-Contreras, Raisa Bu-Coifiu-Fanego, Hermes Fundora Hernández, Jesús Callol Barroso, Consuelo Sanchez-Martinez

**Affiliations:** 1 Central laboratory of CSF (LABCEL), Faculty of Medical Sciences "Dr Miguel Enríquez", Superior Institute of Medical Sciences of Havana, Section 10049, 11000 CP Havana City, Cuba

## Abstract

**Background:**

Eosinophilic meningoencephalitis caused by the helminth *Angiostrongylus cantonensis*, is an emerging infectious disease in America. The objective of this paper was to determine if the intrathecal synthesis of immunoglobulin E is produced during the acute phase of the disease.

**Methods:**

Thirteen patients, mean age 4.5 years were studied; a diagnostic lumbar puncture was performed and serum samples taken. Immunoglobulin E (IgE) in serum and in cerebrospinal fluid (CSF) was quantified by nephelometry. Control patients had other infections or other neurological diseases.

**Results:**

The mean cell count in the CSF was 500 × 10^-6 ^cells/L and of these 23% were eosinophils. In blood the eosinophils were 13%. The chief symptoms of the patients were migraine, vomiting and fever and 50% presented some meningeal signs. IgE intrathecal synthesis analyzed by the corresponding quotient diagram (Reibergram) was observed in all patients. No intrathecal IgE synthesis was seen in control patients.

**Conclusion:**

Intrathecal synthesis of IgE demonstrates the participation of this immunoglobulin in the destruction of the third stage larvae of the parasite in the CSF. The test should be considered in our environment as a tool to aid diagnosis.

## Background

Eosinophilic meningoencephalitis is a disease caused by the helminth *Angiostrongylus cantonensis*. The definitive hosts of this parasite are rats, *Rattus rattus *and *Rattus norvegicus *[1-3]. Many species of mollusc constitute intermediary hosts [4] and are responsible for the transmission of this zoonosis. Eosinophilic meningoencephalitis described for the first time in Southeast Asia and later in Asia, Africa and the Caribbean [5-8], is considered an important and sometimes fatal human disease. In Cuba this disease is primarily observed in children with a mild course, because the number of larvae accidentally ingested is small. Since 1981, when in the Paediatric Hospital of San Miguel del Padron, the first case was observed in the Americas, an average of 3 cases per year have been reported.

IgE plays an important role in anaphylactic type 1-hypersensitivity mechanisms, with high values in patients with parasitic infectious diseases accompanied by eosinophilia [5].

In an earlier study [9] on four patients diagnosed with eosinophilic meningoencephalitis caused by *Angiostrongylus cantonensis*, it was shown that a local IgE synthesis was detected in the first diagnostic lumbar puncture without intrathecal synthesis of immunoglobulin A (IgA), immunoglobulin M (IgM) or immunoglobulin G (IgG). In the present study, we investigated intrathecal IgE synthesis in a larger group of children using CSF/serum quotient diagrams (Reibergrams) and report here an extremely high intrathecal immune response.

The novelty of these results is that IgE intrathecal synthesis may be considered in our environment as a diagnostic tool, and as an auxiliary diagnostic method in countries, having other parasites that are involved with the central nervous system.

## Methods

### Patients and sample collection

Thirteen patients of an average age of 4.5 years with a diagnosis of eosinophilic meningoencephalitis caused by *Angiostrongylus cantonensis *were studied. The patients were admitted to the Pediatric Hospital of San Miguel Padrón, of the City of Havana between 2002 and 2007 with typical symptoms. CSF and serum samples from 31 patients with other non-neurological infections and other neurological disorders were used as controls. Normally there are no eosinophils in CSF and the blood eosinophil count was expected to be normal in this control group. The project was approved by the hospital ethical committee and written informed consent of the parent or guardian was obtained. CSF lumbar puncture and blood extraction were performed. Because samples were taken for routine analysis, no extra volume was needed for IgE analysis. Samples were collected at the time of admission at the onset of the symptoms, and were kept in small aliquots at -80°C until analysis.

### Analysis of CSF and serum

CSF and serum total and differential cell counts were performed by traditional methods. The levels of IgE in serum and CSF were quantified by N Latex IgE Mono assay in a BN Prospec nephelometer (Dade Behring, Marburg, Germany). The procedure was standardized for the quantification of IgE in both fluids and enabled the detection of intrathecal synthesis of IgE. Serum albumin was quantified using NOR Partigen radial immunodiffusion plates and in CSF using LC Partigen plates (Dade Behring, Marburg Germany). The data for albumin gave an estimate of the integrity of the blood-CSF barrier.

### Data analysis

**Figure 1:**
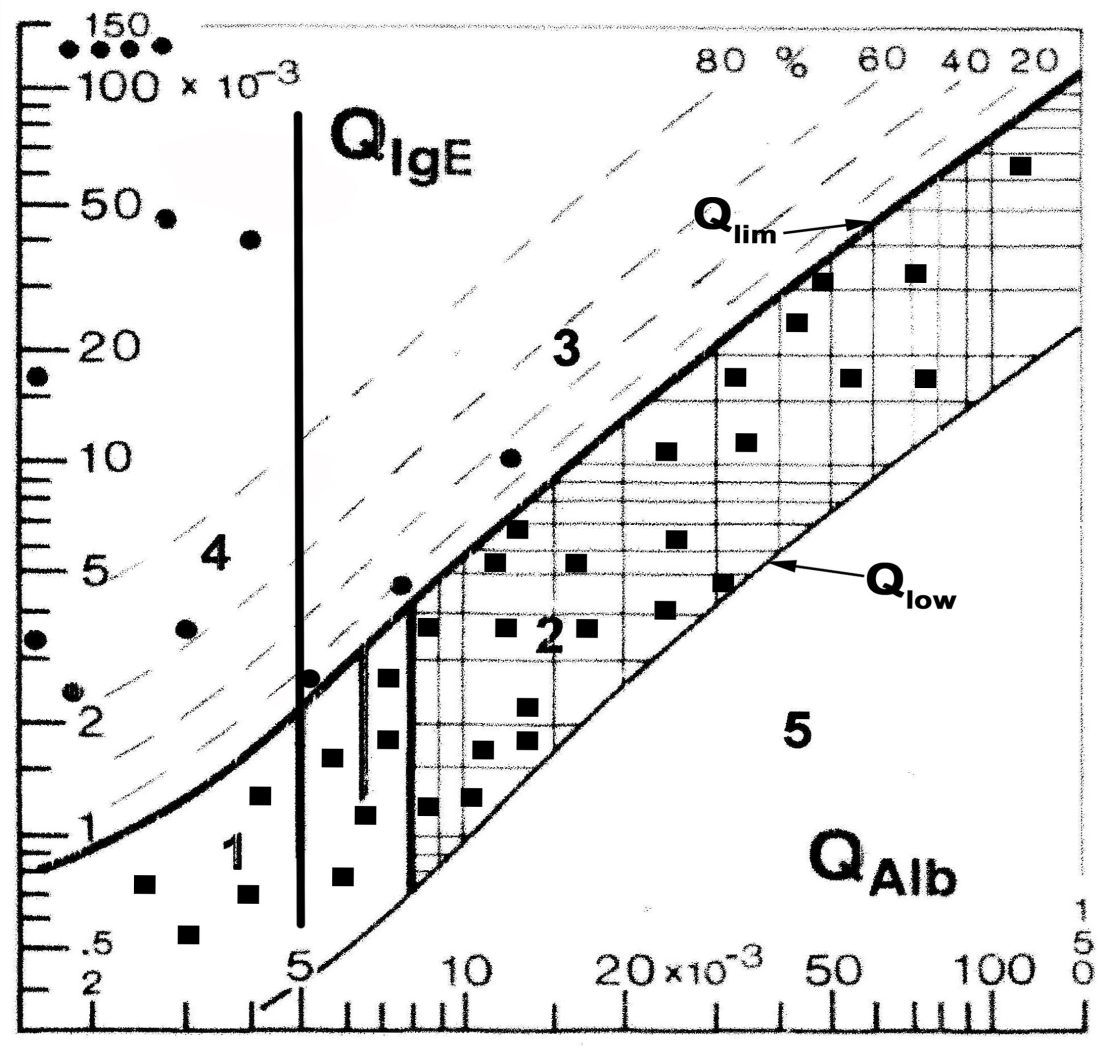
CSF/serum quotient diagram for IgE (Reibergram) depicting the intrathecal synthesis of IgE in children with eosinophilic meningoencephalitis caused by *Angiostrongylus cantonensis*. The reference ranges for the normal CSF/serum ratios are depicted by the thicker upper hyperbolic line (Q lim) and the thinner lower hyperbolic line (Q low). Values above Q lim represent intrathecal IgE synthesis. The dashed lines indicate the extent of intrathecal synthesis as intrathecal fractions (IgE IF) as 20, 40, 60, and 80% of the measured total immunoglobulin concentration in CSF. The limit of the reference range for Q Albumin due to blood-CSF barrier dysfunction is indicated by three vertical lines for different ages: up to 15 years, up to 40 years, and up to 60 years. The diagram depicts the following ranges: 1, normal; 2, blood-CSF barrier dysfunction (i.e. reduced CSF turnover); 4, intrathecal immunoglobulin synthesis with no change in CSF turnover; 3, intrathecal immunoglobulin synthesis with reduced CSF turnover. Values below the lower hyperbolic line in range 5 would indicate a methodological fault. All the patients (filled circles) have intrathecal synthesis of IgE. The control samples (filled squares) were in the normal range indicating no IgE intrathecal synthesis.

The results were plotted on a quotient diagram (Reibergram) designed for IgE, which was recently introduced for the quantification of the intrathecal IgE synthesis and constructed on the same principle as quotient diagrams for other CSF immunoglobulins [10]. This formula is based on the molecular diffusion theory/CSF flow, the fundamental principle of which is that a decrease in the flow rate is always accompanied by an increase in molecular diffusion from blood to CSF [11-13]. The Reibergram (Fig. 1) was constructed using the ratios Q albumin (CSF albumin/serum albumin) plotted on the ordinate axis and the Q IgE (CSF IgE/serum IgE) on the abscissa axis [9,14]. The axes use logarithmic scales that cover the most frequent ranges for these proteins: 1.5 - 150 × 10^-3 ^for Q Albumin and 0.3 - 150 × 10^-3 ^for Q IgE. The reference ranges for the normal CSF/serum ratios are depicted by the thicker upper hyperbolic line (Q lim) and the thinner lower hyperbolic line (Q low). Between these lines is zone of normal biological variation for Q IgE. Intrathecal synthesis of immunoglobulin is detected when the point falls above the thick hyperbolic line marking the Q boundary (Q limit = 0%). Dashed hyperbolic lines show the magnitude of the intrathecally synthesized fraction relative to the Q limit (IF = 20, 40, 60, 80%).

## Results

From the medical records, the main symptoms were fever and vomiting together with some meningeal signs in 50% of patients. No third stage larvae of the helminth were recovered from the CSF samples. Cerebral angiostrongyliasis is generally diagnosed from the clinical history of the patient and CSF eosinophilia, supported by possible exposure to the infective larvae. The gold standard in the diagnosis remains the detection of the agent that causes the disease, although this is often difficult.

In this study, the average number of cells found in the CSF of patients with the diagnosis of *Angiostrongylus cantonensis *was 500 × 10^-6 ^cells/L with 23% identified as eosinophils. The average proportion of eosinophils in blood was 13%. The quotient diagram, or Reibergram, showed that intrathecal synthesis of IgE was observed for all 13 patients (Fig. 1). Seven patients registered very high IgE intrathecal fractions > 80%, three patients had fractions that were > 60%, one was > 20% and two were > 10%. All results above the 10th percentile (not shown) demonstrate local IgE production. Although IgE intrathecal synthesis is not specific for *Angiostrongylus cantonensis*, IgE synthesis should always be present in eosinophilic meningoencephalitis caused by the larvae of this parasite.

None of the 31 control patients had eosinophils in CSF. Normal CSF cell counts were found in 19 of these and the others had a slightly increased cell count with lymphocyte predominance. The Q IgE values for control patients were plotted (Fig. 1) and all results were between Q lim and Q low, in the zone of normal biological variation for Q IgE.

## Discussion

The patients in this study came from the Municipality of San Miguel Padrón, in the City of Havana. This consists of 150,000 inhabitants, is on the periphery of the Cuban capital, and has rural and semi rural areas. All the affected children lived in houses with earth patios where it is common to also find terrestrial snails and rats, which are, respectively, the intermediate and definitive hosts of the parasite. From 1981, the Pediatric Hospital San Miguel received all the patients from the municipality; a total of 90 patients with an historical incidence of 2 – 3 cases per year.

Although the number of patients affected with the disease is low, this zoonosis is important in our environment. In our sample, we studied patients affected between 2002 and 2007, the diagnosis being by the presence of eosinophils in CSF and clinical symptoms such as fever, vomiting and headache. In Cuba there is no other parasite that can cause eosinophilia in the CSF. Infection with *A. cantonensis *produces a prominent intrathecal production of IgM, IgA and IgG seven to ten days after the beginning of symptoms as we have reported earlier [15]. In this study using the first diagnostic lumbar puncture, IgE intrathecal synthesis was demonstrated but not IgM, IgA or IgG synthesis.

In comparison with a previous paper, when we reported four patients [9], in this study the Q IgE values were less than 200 × 10^-3^. Nevertheless, intrathecal synthesis of IgE was demonstrated in both groups of patients. The average number of cells found in the CSF is slightly different from those first reported [9]. However, these differences do not affect the overall conclusions. Patients received sympt**o**matic treatment only, because they have a non severe meningoencephalitis and antiparasitic treatments were not given. No deaths in children have been reported since the first report in 1981. Most children recovered in 1–3 weeks and the follow-up review showed no sequel in the short term.

IgE displays several properties that differentiate it from the other immunoglobulin classes; among them is a cytotropic capacity. This property increases its average half life in the blood by 3 to 4 fold when it is attached to specific receptors on mastocytes and basophils. These cells are involved in the allergic processes, and this is often thought to be their main function. However, macrophages, eosinophils and platelets also have receptors for IgE, which explains IgE participation in the defense against helminths [16,17], particularly *Angiostrongylus cantonensis*. This defense is mediated by IgE antibodies and eosinophils, a special type of antibody dependent cell-mediated cytotoxicity (ADCC), in which the IgE antibodies bind to the surface of the helminths through receptors, activating the eosinophils to secrete enzymes that destroy the parasites [16,17]. The IgE has an average lifetime of three days and has an important role, due to its interaction with the larvae of the third stage of this parasitosis, causing the liberation of neurotoxins responsible for the inflammatory process [18]. There is evidence from other research groups that IgE is involved in the CNS immune response in this disease [19,20]. This is supported by the large amount of intrathecally synthesized IgE observed in this study.

In our experience, IgE in the CSF and serum in eosinophilic meningoencephalitis caused by *Angiostrongylus cantonensis *is the immunoglobulin that shows the largest increase compared to IgA, IgG and IgM. Consequently, intrathecal synthesis of this immunoglobulin can contribute to confirmation of the diagnosis, along with clinical symptoms, and can be an early marker of this neuroparasitosis.

The advantage of using the Reibergram for demonstrating IgE intrathecal synthesis is that it shows the fraction of IgE produced in the central nervous system, indicating the local response to the *Angiostrongylus cantonensis *third stage larvae. This method takes into account IgE that enters the CSF from the serum and shows the additional amount found in the CSF due to local production.

In Cuba, there is evidence that no other parasites cause eosinophilic meningoencephalitis, which makes the intrathecal synthesis of IgE a diagnostic tool for research in our environment [21,22]. From a clinical perspective, measuring IgE for routine diagnosis is not practical for general practice because of the additional cost.

## Conclusion

IgE synthesis has an essential role in inflammatory processes of the eosinophilic meningoencephalitis caused by *Angiostrongylus cantonensis *and should be considered as a diagnostic tool. In other countries where it is common to find other parasites in the CSF as for example in neurocysticercosis, the diagnostic value of finding IgE intrathecal synthesis is more limited and would only be an indicator of an eosinophilic meningoencephalitis of parasitic origin.

## Competing interests

The authors declare that they have no competing interests.

## Authors' contributions

BPD and AJDC designed the study, performed coordination and drafted the manuscript. RBCF participate in its design and review the clinical profiles of patients. HFH helped to measure protein analysis and to draft the manuscript. JCB contributed to the manuscript. CSM participated in the laboratory coordination and contributed to the manuscript. All authors read and approved the final version of the manuscript.
